# High-fidelity parameter-efficient fine-tuning for joint recognition and linking of diagnoses to ICD-10 in non-standard primary care notes

**DOI:** 10.1093/jamiaopen/ooaf120

**Published:** 2025-10-16

**Authors:** Cristian Estupiñán-Ojeda, Raúl J Sandomingo-Freire, Lluís Padró, Jordi Turmo

**Affiliations:** SIANI, University of Las Palmas de Gran Canaria, Las Palmas de Gran Canaria 35017, Spain; Intelligent Data Science and Artificial Intelligence Research Group (IDEAI-UPC), Universitat Politècnica de Catalunya, Barcelona 08034, Spain; Intelligent Data Science and Artificial Intelligence Research Group (IDEAI-UPC), Universitat Politècnica de Catalunya, Barcelona 08034, Spain; Intelligent Data Science and Artificial Intelligence Research Group (IDEAI-UPC), Universitat Politècnica de Catalunya, Barcelona 08034, Spain

**Keywords:** natural language processing, joint entity recognition and linking, ICD-10 codes, parameter-efficient fine-tuning

## Abstract

**Objectives:**

Joint recognition and ICD-10 linking of diagnoses in bilingual, non-standard Spanish and Catalan primary care notes is challenging. We evaluate parameter-efficient fine-tuning (PEFT) techniques as a resource-conscious alternative to full fine-tuning (FFT) for multi-label clinical text classification.

**Materials and Methods:**

On a corpus of 21 812 Catalan and Spanish clinical notes from Catalonia, we compared the PEFT techniques LoRA, DoRA, LoHA, LoKR, and QLoRA applied to multilingual transformers (BERT, RoBERTa, DistilBERT, and mDeBERTa).

**Results:**

FFT delivered the best strict Micro-F1 (63.0), but BERT-QLoRA scored 62.2, only 0.8 points lower, while reducing trainable parameters by 67.5% and memory by 33.7%. Training on combined bilingual data consistently improved generalization across individual languages.

**Discussion:**

The small FFT margin was confined to rare labels, indicating limited benefit from updating all parameters. Among PEFT techniques, QLoRA offered the strongest accuracy-efficiency balance; LoRA and DoRA were competitive, whereas LoHA and LoKR incurred larger losses. Adapter rank mattered: ranks below 128 sharply degraded Micro-F1. The substantial memory savings enable deployment on commodity GPUs while delivering performance very close to FFT.

**Conclusion:**

PEFT, particularly QLoRA, supports accurate and memory-efficient joint entity recognition and ICD-10 linking in multilingual, low-resource clinical settings.

## Introduction

The integration of artificial intelligence (AI) into healthcare, particularly in primary care settings, has the potential to transform clinical decision making by improving recognition and linking of medical diagnoses. This study focuses on the application of parameter-efficient fine-tuning (PEFT) methods for joint entity recognition and linking (JERL) using ICD-10 codes, specifically on a dataset that closely reflects real-world challenges. The UPC-IDIAP dataset, characterized by non-standard language use and bilingual content in Catalan and Spanish (IDIAP data and UPC annotations), presents significant challenges due to its inherent noise and unbalanced distribution of diagnoses. Unlike standard benchmark datasets, state-of-the-art challenges do not address this type of nonstandard clinical text, making the task fundamentally different. These characteristics make it a relevant context in which to evaluate advanced techniques for accurately processing and analyzing such complex data.

Previous approaches to entity recognition and linking in clinical texts rely on fully parameterized models, which, while effective, require considerable computational resources and memory. This reliance may create a bottleneck, as training these models becomes prohibitively expensive in settings where computational power is a limitation, leading to slower adoption of AI tools. Traditional fine-tuning methods, which update all model parameters regardless of task-specific needs, end up with redundant computational overhead. Addressing this inefficiency is urgent, as scalable AI solutions are nowadays essential to democratize access to advanced diagnostics, particularly in low-resource primary care settings. Failure to optimize these models risks perpetuating inequalities in healthcare quality and accessibility. This study explores the potential of PEFT methods, in particular LoRA, DoRA, LoHA, LoKR, and QLoRA, as a more resource-efficient alternative. These methods aim to maintain high performance while drastically reducing the number of trainable parameters, making them particularly suitable for resource-constrained environments.

By adapting the models to the specific linguistic and medical characteristics of the dataset, this research aims to improve the accuracy and efficiency of diagnostic coding in primary care. The results not only highlight the effectiveness of PEFT models in processing complex clinical data but also underscore their potential for wider application in multilingual healthcare environments, paving the way for more accessible and scalable AI-based diagnostic tools.

## Background and significance

The JERL task for ICD-10 codes combines the challenges of named entity recognition (NER) and entity linking (EL), which have traditionally been approached as separate tasks. It is to note that terminology may be somewhat confusing: terms such as “normalizing,” “linking,” and “grounding” are often used interchangeably. In addition, some papers use “entity linking” to encompass the whole recognition and linking process, whereas others denote only the second part.^1^ In the text, we will use “linking,” and in doing so, we will refer only to the second step. With respect to the biomedical domain, the second step is sometimes also referred to by acronyms like BNEN (biomedical entity normalization)^2^ and BEL (biomedical entity linking),^1^ whereas in medical settings the reader may come across with abbreviations such as MER (medical entity recognition) and MEN (medical entity normalization).^3^

Recent studies have demonstrated the benefits of integrating these 2 tasks, to enhance performance and generalizability. Martins et al^4^ followed this approach, showing that training the NER and EL models together produces superior results compared to models trained individually. Their model, inspired by the Stack-LSTM approach, achieved competitive results across both tasks, highlighting the interdependency between entity recognition and linking. Zhao et al^3^ pioneered a multi-task learning scheme to address disease JERL: one task took care of the entity recognition, while the other task dealt with linking. They jointly modeled both of them by setting an explicit feedback mechanism between them. In other words, the result of NER was fed into the input of EL and vice versa, thus converting 2 hierarchical tasks into a 2-task parallel scheme. They proved several deep learning architectures and the results improved state-of-the-art in 2 publicly available datasets.

Biomedical datasets like MedMentions^5^ and MIMIC-IV^6^ have been instrumental in advancing research in this area. MedMentions provides a large corpus annotated with UMLS concepts, serving as a robust resource for NER and EL in the biomedical field. MIMIC-IV, a comprehensive electronic health record dataset, offers a rich source of clinical notes. However, most real-world electronic health records (EHR) datasets are affected by significant noise, including mention imbalances, which distinguish them from more curated datasets like MIMIC-IV or MedMentions. This noise, coupled with severe overall class imbalance, poses substantial challenges for standard machine learning models, often leading to reduced generalization. Given that the dataset involved in this study also exhibits these inherent issues, it is essential to recognize these challenges as they may impact the accuracy and reliability of the findings.^7^^,^^8^ Transformer-based models have shown promise in entity linking tasks. López-García et al^9^ demonstrated the efficacy of in-domain adapted transformers trained on clinical notes in Spanish, achieving state-of-the-art performance in tasks requiring both entity recognition and linking.

The importance of fine-tuning pre-trained models on domain-specific data has been underscored in several studies. Gligic et al^10^ used transfer learning to enhance NER in electronic health records, achieving significant improvements by pre-training on large, unannotated corpora and fine-tuning on specific tasks.

Entity linking, particularly in medical contexts, has also seen advances. Yan et al^11^ introduced an unsupervised entity linking model using multi-instance learning (MIL) to improve the accuracy of linking Chinese medical symptom mentions to the ICD-10 classification. Also, Noh and Kavuluru^12^ leveraged a SciBERT model to jointly optimize biomedical NER and entity linking. Although their work advanced JERL for biomedical texts, it mainly focused on structured scientific literature (MedMentions dataset) rather than noisy, bilingual clinical notes like those in primary care settings. They also did not concentrate on ICD diagnoses, but on a wide range of medical entities, including also drugs and genes.

Building on these foundations, recent work has begun to leverage prompt-based learning and candidate-level interaction mechanisms to address long-standing challenges in biomedical entity linking. In contrast to traditional re-ranking models, which typically evaluate each candidate separately alongside the mention context, the approach proposed by Xu et al^13^ processes all candidates jointly. Their method achieves strong results along 3 benchmark datasets—NCBI disease, BC5CDR, and COMETA—demonstrating the value of incorporating both contextual and inter-candidate information.

A complementary strategy is proposed by Zhu et al,^14^ who introduce a 2-phase linking pipeline. The first phase employs a bi-encoder to generate candidate entities efficiently, while the second applies a more refined, prompt-based re-ranking stage that makes use of contextual cues in the surrounding text. Their system shows consistent performance gains over prior methods when evaluated on MedMentions and the NCBI disease corpus. Collectively, these studies reflect a broader trend towards using lightweight, flexible mechanisms like prompt tuning to improve entity linking in complex and noisy clinical text environments.

Moreover, the emergence of PEFT techniques, although primarily explored in tasks such as clinical diagnosis prediction as in the CPLLM model by Ben Shoham and Rappoport,^15^ presents a promising avenue for optimizing large language models in various clinical applications.

## Materials and methods

### Objective

The objective of the study is to evaluate which of 4 models has the best results in JERL of ICD-10 codes from non-standard bilingual primary care clinical notes. We aim to use PEFT to improve the utilization of computational resources, so that healthcare professionals can efficiently and accurately identify diseases in a manner that reduce computational costs. Starting from the development of an improved diagnostic mapping system for medical records to ICD-10 codes, this research aims to help clinical practitioners make more informed decisions and, therefore, more personalized and efficient healthcare in a variety of primary care settings. This will ultimately contribute to better patient outcomes and resource utilization.

### Data

The UPC-IDIAP dataset utilized in this project comprises clinical notes authored by primary care physicians in Catalonia during patient visits. It spans 6 years and includes 21 812 de-identified notes from 320 multimorbid patients aged over 50 with heart disease or stroke, with each note corresponding to a single visit. On average, there are 68.16 visits per patient ranging from 1 to 174. The length of these documents varies from a few tokens to 620 tokens, although most are under 40 tokens. In total, the dataset contains 1 008 548 tokens. This dataset was generously provided by the Primary Healthcare Research Foundation IDIAP JGol.^16^ Due to the sensitive nature of the data and the conditions under which it was provided, the UPC-IDIAP dataset is not publicly available.

These notes are written in a mix of non-standard Spanish and Catalan, featuring elisions, both standard and non-standard abbreviations, typos, and a blend of both languages, among other distortions, which increase the difficulty of the task. This linguistic variability, along with the informal and unstructured nature of the notes, contrasts sharply with standard clinical corpora used in state-of-the-art benchmarks, which are typically monolingual, standardized, and more formally written. Therefore, the tasks addressed with this dataset require methods capable of handling noisy, code-switched, and domain-specific language, posing unique challenges rarely covered by existing benchmarks. Some examples of these linguistic phenomena are shown in Table 1.

**Table 1.** Extracts from our bilingual Spanish/Catalan clinical notes.

Language	Example	Approximate translation
Spanish	M: Descartar TBC. ECAR.2139 .E: Pt anterior positiva. Cicatriz de B.C.G. No contacto conocido de TBC.	*S: Discard TBC. ECAR.2139 .O: positive anterior Pt. B.C.G Scar. No known TBC contact.*
Catalan	E: 10/2016 RMN Cervical uncodiscartr segm C2-7 q condiciona estenosi foraminal segm C3-7 amb abombaments discals i hipertrof llig grocs associada q condicionen estenosi de canal al mateix segment. …	*O: 10/2016 MRI Cervical spine uncodiscartr segm C2-7 conditioning foraminal stenosis segm C3-7 with disc bulges and associated yellow lig hypertroph conditioning canal stenosis at the same segment. …*
Catalan with Spanish	… TOS RESIDUAL PERÒ MILLORADA de l’ofec, ”me encuentro bien ahora”. … canvis crònics a bases, cardiomegàlia aprox. ICT 55% No ha aconseguit recollir mostres d’esput .E: TA 138/90 .A: ASMA BRONQUIAL .P: singulair 10 crònic + aerosols si calgués en exacerb. - ecocardio	*… RESIDUAL COUGH BUT IMPROVED from shortness of breath, ”I feel fine now”. … chronic changes in bases, approx. cardiomegaly. ICT 55% he couldn’t collect sputum samples .O: BP 138/90 .A: BRONCHIAL ASTHMA .P: chronic singulair 10 + aerosols if needed during exacerb. - echocardio*

The English translation tries to reflect the use of non-standard language in the original.

Typically, each clinical note is expected to adhere to the *SOAP* structure, comprising 4 sections that align with the steps a doctor follows during a visit: **S**ubjective, **O**bjective, **A**ssessment, and **P**lan. However, this structure is largely inconsistent within our dataset, and even when present, the sections are often indistinct. Consequently, we treated the notes as free-text documents.

Three experts in clinical-annotation from IDIAP annotated the corpus under established medical guidelines. Each expert annotated all the documents. The initial agreement among the 3 annotators was 77.8%. Disagreements and edge-cases were resolved internally. Authors only performed data preprocessing (eg document-structuring, language identification [Spanish/Catalan], case-normalization).

Manual annotation identified 4 types of medical entities mentioned in the clinical notes (diagnoses, signs/symptoms, drugs and body parts) and several types of relationships between entities (eg coadministration and replacement between 2 drugs, causality between a diagnosis or a sign/symptom and a diagnosis, location between a diagnosis and a body part). Each entity mention was labeled with a list of properties including its corresponding code from standard medical codings: ICD-10 for diagnoses, ICPC-2 for signs/symptoms, ATC-7 for drugs, and SNOMED-CT for body parts.

**Figure 1.**
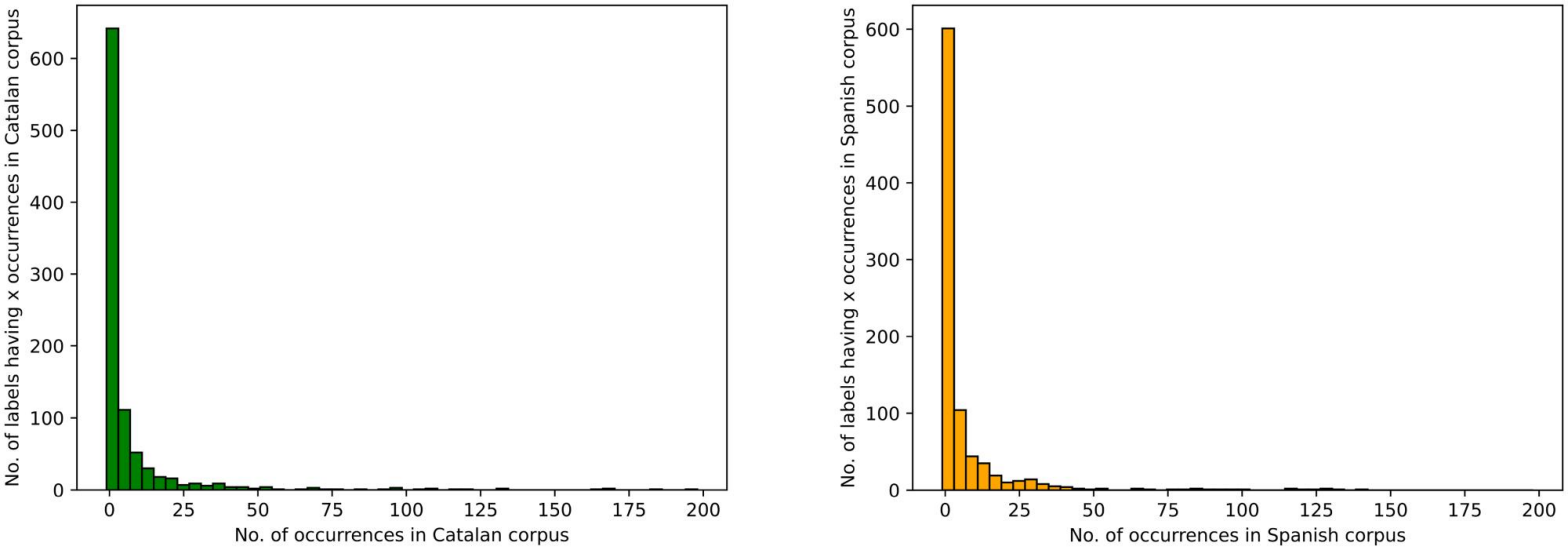
Label distribution across Catalan (left) and Spanish (right) clinical documents. Each column represents an interval of 4 mentions (ie the first column in each diagram encompasses labels having 1-4 annotated mentions). Both languages share the same pattern: an unbalanced long-tailed distribution where the vast majority of labels have a handful of mentions, although labels with dozens or even hundreds of annotations do exist.

In this study, we specifically focused on the detection and ICD-10 linking of the diagnoses mentioned in the clinical notes. In total, 16 000 diagnoses were annotated. As shown in Figure 1, the distribution of these annotations was similar for both Catalan and Spanish: 8647 and 7353, respectively.

### Parameter-efficient fine-tuning

PEFT in NLP leverages pre-trained language models by fine-tuning a smaller, task-specific subset of parameters while keeping the majority fixed. This significantly reduces computational costs, making it ideal for resource-constrained environments such as ICD-10 disease classification. PEFT ensures high accuracy in clinical settings where computational resources are limited, enhancing healthcare data management and patient care.

We explore 3 key PEFT techniques:

- **LoRA**: Low-Rank Adaptation (LoRA) technique focuses on updating a new low-rank subset of weights rather than the existing weights set, leading to more efficient parameter training. It strikes a balance between efficiency and accuracy by training less parameters than the original model.^17^
- **DoRA**: By decomposing weights into direction and magnitude, DoRA refines the adaptation process, stabilizing training and enhancing performance, especially when dealing with lower rank sizes. This method is particularly useful for scenarios that require high stability and precision.^18^
- **LoHA**: Low-Rank Hadamard Product (LoHA) is a variant of Low-Rank Adaptation (LoRA) that approximates large weight matrices using multiple low-rank matrices combined via the Hadamard product. This approach enhances parameter efficiency while achieving performance comparable to standard LoRA methods.^19^
- **LoKR**: Low-Rank Kronecker Product (LoKR) is a technique that utilizes the Kronecker product to combine low-rank matrices for model adaptation. By leveraging the Kronecker product, LoKR captures complex interactions within the model parameters, facilitating efficient and effective fine-tuning.^20^
- **QLoRA**: Integrating quantization with LoRA, QLoRA reduces model precision to decrease memory usage and computational requirements, making it especially effective for very large models. Despite reduced precision, QLoRA maintains performance levels, crucial for large-scale clinical applications.^21^

### Models

In this study, we used a range of language models designed to meet the unique linguistic and domain-specific needs of our dataset.

- **BERT Base Multilingual Cased (BERT):** Pre-trained on 104 languages, including Spanish and Catalan, using a masked language modeling objective. This model’s robust multilingual capabilities are essential for interpreting clinical notes containing mixed languages, ensuring accurate entity recognition and linking.^22^
- **DistilBERT Base Multilingual Cased (DistilBERT):** This distilled version of BERT retains much of the original model’s performance while being smaller and faster. It is particularly well-suited for processing large volumes of clinical notes efficiently, without sacrificing accuracy.^23^
- **mDeBERTa V3 Base (mDeBERTa):** A multilingual version of DeBERTa developed by Microsoft, utilizing the same architecture as DeBERTa and trained with multilingual data from CC100. This model was trained using 2.5 trillion tokens from the CC100 dataset, making it highly proficient in natural language understanding tasks across multiple languages.^24^
- **RoBERTa Es WikiCAT Es (RoBERTa):** Pre-trained using texts from the Spanish National Library and WikiCAT, this model is fine-tuned for Spanish and Catalan text classification. Developed by the Barcelona Supercomputing Center, it excels in handling the bilingual nature of our dataset.

### Hyperparameters

We employed a comprehensive grid search strategy for hyperparameter optimization, essential to ensure that our models achieved stable and reliable performance. The hyperparameters considered include:

- Number of training epochs: fixed at 50.
- Batch size: values 4, 8, and 16 were tested.
- Learning rate: A range of values between 1e-04 and 2e-06 was explored.
- Learning rate scheduler: an Exponential Learning Rate Scheduler was used, with a gamma value tested in the range of 0.50 to 0.99.
- Rank size for PEFT models: Values of 8, 16, 32, 64, 128, and 256 were tested.
- Alpha parameter for PEFT: Values of 64, 128, 256, 512, 1024, and 2048 were tested.
- Dropout rate: Values of 0.0, 0.1, 0.3, and 0.5 were tested.
- Quantization precision in QLoRA: Fixed at 4-bit NormalFloat (NF4).
- Compute data type in QLoRA: Fixed at bfloat16.

These values were carefully selected and fine-tuned based on the specific characteristics of the dataset and the need for stable convergence in training. The final values for these hyperparameters and the optimal combinations will be discussed in Results section.

### Mention preprocess

For preprocessing, we used the BIO tagging technique. In this method, “B-ZZ.Z” marks the beginning of a mention with code “ZZ.Z,” which corresponds to one of the possible ICD-10 codes, “I-ZZ.Z” continues the mention, and “O” marks the remaining segments of the diagnosis that are not of interest. This tagging ensures the precise identification of ICD-10 mentions within clinical notes.

**Figure 2.**
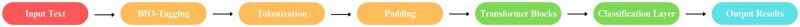
The processing and training flow of the architectures, illustrating steps from input text through BIO-tagging, tokenization, padding and special tokens handling, transformer blocks, linear classification layer, to the output results.

The text was then tokenized, padded, and converted into numerical identifiers, with special tokens and padding labeled “X” (numerical value of -100) to be ignored during the loss calculation. When words split into multiple tokens, the first token receives the BIO tag, while subsequent tokens are labeled as “X.” The output from the transformer blocks is finally mapped to ICD-10 mentions through a linear classification layer. The processing flow is depicted in Figure 2.

### Evaluation

Baseline models, including BERT, DistilBERT, mDeBERTa, and RoBERTa, were trained without PEFT optimizations to serve as comparisons. The dataset was partitioned into training (70%), validation (10%), and test (20%) sets, ensuring a robust evaluation framework. Both effectiveness and efficiency metrics were used, as defined in the following sections. Considering both effectiveness and efficiency, our evaluation approach ensures that the models are optimized for deployment in real-world, resource-constrained clinical settings, providing reliable and scalable solutions for healthcare providers.

#### Effectiveness metrics

- **Precision:** Measures the accuracy of the model in identifying true positives among all recognized entities. It is defined as the ratio of correctly identified entities (true positives) to the total number of entities recognized by the model (true positives + false positives).
  $$\text{Precision}=\frac{\text{True}\;\text{Positives}}{\text{True}\;\text{Positives}+\text{False}\;\text{Positives}}$$
- **Recall:** Also known as sensitivity, it measures the model’s ability to correctly identify all relevant entities in the dataset. It is defined as the ratio of correctly identified entities (true positives) to the total number of actual relevant entities (true positives + false negatives).
  $$\text{Recall}=\frac{\text{True}\;\text{Positives}}{\text{True}\;\text{Positives}+\text{False}\;\text{Negatives}}$$
- **Micro-F1 score:** The primary metric for evaluating model performance, emphasizing exact boundary and entity type matches, crucial for assessing precision in entity recognition. It is defined as the harmonic mean of precision (ratio of correctly bounded and ICD-10-linked diagnoses to the diagnoses recognized by the model) and recall or sensitivity (ratio of correctly bounded and ICD-10 linked diagnoses to the total of diagnoses).
  $$F1_{\mathrm{micro}}=\frac{2\times \text{Precision}\times \text{Recall}}{\text{Precision}+\text{Recall}}$$
- **Macro-F1 score:** Provides additional insight by evaluating performance across all classes, highlighting the model’s effectiveness in handling both frequent and underrepresented entities.
  $$F1_{\mathrm{macro}}=\frac{1}{N}\sum_{i=1}^{N}F1_{i}$$

#### Efficiency metrics

- **Trainable parameters:** Assesses the total number of parameters subject to training, particularly relevant for PEFT methods where minimizing resource usage is critical.
- **Training time:** Measures the total time required for training, crucial for assessing model suitability in real-time clinical environments.
- **Document inference:** Evaluates how quickly the model processes new data, an important factor in time-sensitive applications such as clinical decision support systems.
- **Memory allocated:** Tracks the memory usage of the model during training and inference, ensuring that it can be deployed on systems with limited resources.

## Results

All experiments were conducted using the best hyperparameter configurations obtained through systematic hyperparameter search. Unless otherwise stated, models were trained for 50 epochs with a batch size of 4 and a dropout of 0.2 in the classification head. In the result tables, a dagger symbol (^†^) indicates experiments trained with a batch size of 8, which was selected as optimal in some cases during tuning. This variation should be taken into account when comparing memory usage between models.

Full fine-tuning (FFT) models used a learning rate of 3e-5. All PEFT methods, including LoRA, QLoRA, DoRA, LoHA, and LoKR, were configured with rank r=256, α=2048, and no bias adaptation unless otherwise specified. QLoRA models additionally employed NF4 quantization. LoHA and LoKR models used α=1024 with rank and module dropout set to 0.0.

The results are reported in Tables 2-4, which include strict Micro-F1 and Macro-F1 scores for validation and test sets in 3 settings: joint training on Catalan and Spanish notes, training on Catalan notes only, and training on Spanish notes only. Moreover, we report the number of trainable parameters, training time, inference speed, and GPU memory usage. Joint training consistently led to mutual performance gains compared to training on each language independently.

**Table 2.** Parameter-efficient fine-tuning (PEFT) experiments in primary care notes (Catalan and Spanish).

Model	Experiment	Strict micro-F1				Strict macro-F1		Trainable parameters	Training time	Document inference	Memory allocated
		Val	Test	Test CA	Test ES	Val	Test				
RoBERTa	FFT	62.7	61.2	62.7	59.5	38.0	30.5	146.4M	592.3 min	11.4 docs/s	2976.5 MB
RoBERTa	LoRA	61.7	60.6	61.8	59.1	36.8	29.5	64.8M	644.5 min	11.3 docs/s	2569.6 MB
RoBERTa^†^	DoRA	62.2	60.7	61.4	59.8	35.5	28.9	64.9M	643.5 min	11.2 docs/s	5139.8 MB
RoBERTa	QLoRA	62.9	60.5	62.3	58.2	35.6	28.6	64.8M	750.1 min	11.3 docs/s	2424.3 MB
RoBERTa^†^	LoHA	58.6	57.2	59.0	55.1	33.2	26.4	107.2M	619.4 min	11.3 docs/s	4491.6 MB
RoBERTa^†^	LoKR	44.9	45.2	47.6	42.1	18.7	15.3	22.5M	592.7 min	11.3 docs/s	3521.9 MB
DistilBERT	FFT	61.8	60.4	61.9	58.7	37.2	32.6	157.0M	551.0 min	11.5 docs/s	3151.2 MB
DistilBERT	LoRA	60.9	59.8	61.3	57.9	35.0	29.6	43.5M	613.6 min	11.1 docs/s	1974.8 MB
DistilBERT^†^	DoRA	62.9	61.2	63.1	58.9	37.2	30.3	43.6M	621.9 min	11.0 docs/s	3497.9 MB
DistilBERT	QLoRA	54.8	55.7	56.4	54.7	25.5	22.1	43.5M	670.8 min	11.1 docs/s	1843.6 MB
DistilBERT^†^	LoHA	60.3	58.6	60.6	56.2	35.3	30.2	64.8M	613.5 min	10.9 docs/s	3169.6 MB
DistilBERT^†^	LoKR	51.4	49.9	50.9	48.6	22.5	18.9	22.4M	597.3 min	11.1 docs/s	2682.2 MB
BERT	FFT	64.0	63.0	64.2	61.5	39.1	35.8	199.6M	604.1 min	11.3 docs/s	3992.1 MB
BERT^†^	LoRA	63.1	61.1	61.9	60.0	37.3	30.6	64.8M	598.3 min	11.2 docs/s	4070.5 MB
BERT	DoRA	59.7	57.7	58.5	56.6	31.1	25.0	64.9M	759.4 min	10.7 docs/s	3442.0 MB
BERT	QLoRA	63.5	62.2	62.8	61.5	38.8	32.2	64.8M	765.1 min	11.3 docs/s	2644.5 MB
BERT^†^	LoHA	62.5	60.3	62.2	57.9	40.1	33.1	107.2M	643.7 min	11.2 docs/s	4744.2 MB
BERT^†^	LoKR	53.5	52.4	54.3	50.1	25.9	21.8	22.5M	619.2 min	11.1 docs/s	3771.6 MB
mDeBERTa	FFT	60.7	57.7	59.2	56.0	35.9	27.4	300.5M	662.9 min	11.3 docs/s	5936.2 MB
mDeBERTa	LoRA	59.7	58.5	60.4	56.2	32.5	26.6	64.8M	727.6 min	11.2 docs/s	4163.0 MB
mDeBERTa^†^	DoRA	62.5	59.6	61.8	56.8	36.4	28.3	64.9M	669.7 min	11.1 docs/s	7765.9 MB
mDeBERTa^†^	QLoRA	54.2	51.1	52.4	49.6	24.2	17.2	64.8M	692.9 min	10.9 docs/s	6372.4 MB
mDeBERTa^†^	LoHA	57.6	54.8	56.6	52.7	31.4	25.8	107.2M	657.7 min	11.2 docs/s	7045.7 MB
mDeBERTa^†^	LoKR	44.6	42.5	43.5	41.3	17.8	14.3	22.5M	644.0 min	11.1 docs/s	6065.5 MB

† Models with batch size 8.

**Table 3.** Parameter-efficient fine-tuning (PEFT) experiments in primary care notes (Catalan).

Model	Experiment	Strict micro-F1		Strict macro-F1		Trainable parameters	Training time	Document inference	Memory allocated
		Val	Test	Val	Test				
RoBERTa	FFT	54.4	54.5	31.2	28.0	146.4M	340.3 min	11.6 docs/s	2966.2 MB
RoBERTa	LoRA	53.3	51.8	25.1	20.5	64.8M	395.3 min	11.2 docs/s	2569.6 MB
RoBERTa^†^	DoRA	54.7	53.1	28.1	23.7	64.9M	369.2 min	11.2 docs/s	5137.8 MB
RoBERTa	QLoRA	56.7	55.8	30.0	25.8	64.8M	431.0 min	11.6 docs/s	2424.3 MB
RoBERTa^†^	LoHA	52.3	51.9	28.1	23.3	107.2M	356.8 min	10.9 docs/s	4490.3 MB
RoBERTa^†^	LoKR	39.0	37.5	15.1	12.7	22.5M	342.1 min	11.5 docs/s	3521.9 MB
DistilBERT	FFT	58.5	58.8	34.6	32.3	157.0M	314.9 min	11.8 docs/s	3146.3 MB
DistilBERT	LoRA	47.5	47.0	20.0	17.0	43.5M	351.0 min	11.4 docs/s	1974.8 MB
DistilBERT^†^	DoRA	52.1	51.9	24.9	21.2	43.6M	353.2 min	11.3 docs/s	3492.9 MB
DistilBERT	QLoRA	50.6	48.5	22.0	17.4	43.5M	354.0 min	11.7 docs/s	1843.6 MB
DistilBERT^†^	LoHA	53.0	53.2	29.4	25.9	64.8M	324.5 min	11.6 docs/s	3167.6 MB
DistilBERT^†^	LoKR	47.9	45.0	19.8	17.5	22.4M	312.7 min	11.7 docs/s	2682.2 MB
BERT	FFT	56.8	56.2	35.0	29.2	199.6M	368.4 min	11.2 docs/s	3982.8 MB
BERT^†^	LoRA	48.5	47.1	19.0	15.2	64.8M	364.4 min	11.2 docs/s	4071.5 MB
BERT	DoRA	54.3	52.0	25.9	21.6	64.9M	439.5 min	11.3 docs/s	3442.0 MB
BERT	QLoRA	39.1	36.7	9.4	6.2	64.8M	448.3 min	11.2 docs/s	2644.5 MB
BERT^†^	LoHA	55.4	54.2	32.2	27.9	107.2M	365.3 min	11.3 docs/s	4744.9 MB
BERT^†^	LoKR	45.9	45.2	22.1	18.0	22.5M	345.6 min	11.4 docs/s	3771.6 MB
mDeBERTa	FFT	55.8	53.6	32.6	25.6	300.5M	384.1 min	11.6 docs/s	5915.7 MB
mDeBERTa	LoRA	52.0	50.7	25.9	18.8	64.8M	426.0 min	11.2 docs/s	4163.0 MB
mDeBERTa^†^	DoRA	46.9	45.7	19.5	14.9	64.9M	409.8 min	10.9 docs/s	7768.4 MB
mDeBERTa^†^	QLoRA	44.1	44.7	16.5	13.4	64.8M	388.0 min	11.1 docs/s	6371.7 MB
mDeBERTa^†^	LoHA	47.5	45.7	23.4	17.8	107.2M	385.7 min	11.2 docs/s	7044.4 MB
mDeBERTa^†^	LoKR	34.5	33.3	12.0	9.2	22.5M	373.6 min	11.2 docs/s	6067.5 MB

† Models with batch size 8.

**Table 4.** Parameter-efficient fine-tuning (PEFT) experiments in primary care notes (Spanish).

Model	Experiment	Strict micro-F1		Strict macro-F1		Trainable parameters	Training time	Document inference	Memory allocated
		Val	Test	Val	Test				
RoBERTa	FFT	56.3	52.0	39.0	28.7	146.4M	243.2 min	11.2 docs/s	2976.4 MB
RoBERTa	LoRA	54.2	48.6	31.7	23.9	64.8M	263.1 min	11.3 docs/s	2569.6 MB
RoBERTa^†^	DoRA	52.0	49.7	30.4	21.9	64.9M	260.1 min	11.0 docs/s	5137.6 MB
RoBERTa	QLoRA	45.1	41.5	22.9	15.8	64.8M	294.2 min	11.3 docs/s	2424.2 MB
RoBERTa^†^	LoHA	47.5	45.7	29.7	22.8	107.2M	270.0 min	11.0 docs/s	4495.6 MB
RoBERTa^†^	LoKR	31.0	30.2	13.5	10.0	22.5M	249.6 min	11.2 docs/s	3521.6 MB
DistilBERT	FFT	56.1	50.7	36.1	26.6	157.0M	206.1 min	11.8 docs/s	3151.1 MB
DistilBERT	LoRA	51.2	48.9	29.9	22.4	43.5M	216.2 min	11.7 docs/s	1974.7 MB
DistilBERT^†^	DoRA	53.1	49.9	31.5	24.0	43.6M	213.6 min	11.6 docs/s	3492.7 MB
DistilBERT	QLoRA	44.5	42.3	21.6	15.2	43.5M	235.0 min	11.7 docs/s	1843.6 MB
DistilBERT^†^	LoHA	48.5	48.0	29.8	25.2	64.8M	212.8 min	11.7 docs/s	3168.3 MB
DistilBERT^†^	LoKR	41.8	41.0	19.3	16.0	22.4M	206.1 min	11.8 docs/s	2682.0 MB
BERT	FFT	57.1	54.6	38.9	30.3	199.6M	243.8 min	11.3 docs/s	3992.0 MB
BERT^†^	LoRA	50.3	49.7	27.5	22.5	64.8M	260.1 min	11.2 docs/s	4071.2 MB
BERT	DoRA	47.3	45.8	25.9	19.2	64.9M	312.0 min	10.8 docs/s	3441.9 MB
BERT	QLoRA	54.9	51.9	34.0	25.3	64.8M	303.4 min	11.1 docs/s	2644.4 MB
BERT^†^	LoHA	51.1	47.8	31.5	24.8	107.2M	255.3 min	11.2 docs/s	4744.7 MB
BERT^†^	LoKR	43.0	41.0	20.7	16.1	22.5M	228.9 min	11.5 docs/s	3771.4 MB
mDeBERTa	FFT	51.6	49.5	33.7	23.9	300.5M	254.8 min	11.6 docs/s	5935.1 MB
mDeBERTa	LoRA	32.4	31.3	11.6	8.0	64.8M	292.9 min	11.2 docs/s	4162.9 MB
mDeBERTa^†^	DoRA	42.9	42.8	21.3	16.1	64.9M	257.1 min	11.2 docs/s	7769.2 MB
mDeBERTa^†^	QLoRA	26.3	25.8	8.7	5.3	64.8M	254.0 min	10.9 docs/s	6371.5 MB
mDeBERTa^†^	LoHA	37.4	35.6	18.2	13.2	107.2M	254.1 min	11.2 docs/s	7046.4 MB
mDeBERTa^†^	LoKR	23.7	26.7	8.2	6.7	22.5M	248.0 min	11.4 docs/s	6067.3 MB

† Models with batch size 8.

The model that overall performs best is BERT with FFT, which achieved a strict Micro-F1 of 63.0 on the joint test set. The best PEFT approach is BERT with QLoRA, which reached 62.2, a difference of 0.8 points, or 1.27% relative performance gap. Despite this, QLoRA requires only 64.8M trainable parameters, compared to 199.6M for FFT, representing a 67.5% reduction. Furthermore, QLoRA consumes 2644.5 MB of memory during inference, compared to 3992.1 MB for FFT, a 33.7% reduction.

This small performance gap translates into only 189 missed mentions in total. These mismatches spanned 189 unique labels, whose frequency statistics are summarized below.

Max occurrences per label: 152

Min occurrences: 1

Mean ± standard deviation: 8.76 ± 16.50

Focusing on the difference in correct predictions per label between FFT and QLoRA:

Max difference: 7

Min difference: 1

Mean ± standard deviation: 1.31 ± 0.81

The label with the highest difference was T14.2 (fracture of unspecified body region), where FFT predicted 7 more correct instances than QLoRA.

**Figure 3.**
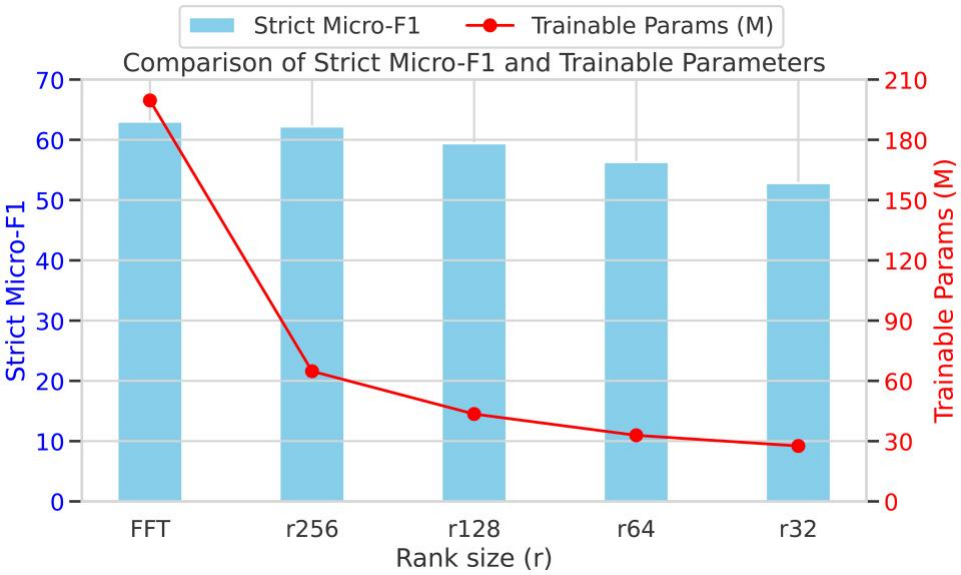
Strict Micro-F1 vs number of trainable parameters for QLoRA with BERT at varying adapter ranks, evaluated on the joint Catalan-Spanish dataset. Lower ranks lead to improved efficiency at the cost of performance.

To quantify the parameter-performance trade-off, we varied QLoRA rank *r* in BERT: FFT gives 63.0 Strict Micro-F1 with 199.6 M parameters and 4 GB memory; rank 256 reaches 62.2/64.8 M/2.6 GB, rank 128 59.4/43.5 M/2.3 GB, rank 64 56.3/32.9 M/2.2 GB, and rank 32 52.8/27.6 M/2.2 GB. Smaller ranks save resources but erode accuracy, as shown in Figure 3.

The lowest memory footprint was obtained by QLoRA with BERT at rank 32, which required only 2155 MB during inference. However, this comes at a significant performance cost, with strict Micro-F1 dropping to 52.8, more than 10 points below the FFT baseline.

## Discussion

The results presented above provide a concise evaluation of PEFT for Joint Entity Recognition and Linking (JERL) in Catalan and Spanish non-standard primary-care notes.

### FFT vs PEFT approaches

FFT with BERT achieved the best strict Micro-F1 (63.0) with 199.6M trainable parameters and ≈4GB inference memory. BERT-QLoRA (rank 256) reached 62.2, only 0.8 points lower, while cutting trainable parameters by >66% and memory by 33.7%. The extra 189 correct FFT predictions were dispersed across low-frequency labels; the largest individual gain (7 instances) concerned “T14.2: Fracture of unspecified body region.” Thus, FFT’s margin is confined to the rare tail of the label distribution rather than widespread across common entity types.

### Effectiveness and limits of Adapter-Based methods

QLoRA offered the most robust gains, whereas LoRA and DoRA stayed within 1-2 points on several bases (eg RoBERTa, DistilBERT). By contrast, LoHA and especially LoKR degraded performance sharply (eg BERT-LoKR 45.2), failing to exchange accuracy for additional efficiency; their rank-aware projections appear unable to capture the fine-grained context JERL requires.

### Impact of language composition

Joint Catalan-Spanish training consistently benefited FFT, QLoRA, and DoRA, exploiting the languages’ lexical proximity. Yet aggressive compression (LoKR or QLoRA rank 32) sometimes harmed cross-lingual transfer, indicating that adapter capacity must balance parameter savings with representational breadth.

### Rank sensitivity in QLoRA

Reducing QLoRA rank exposed a clear efficiency-accuracy trade-off: dropping from 256 to 128 cost 2.8 F1, and to 32 cost 9.4, while nearly halving parameters and trimming several hundred MB of memory. Complex, token-level tasks like JERL therefore still demand relatively high ranks.

### Implications for clinical NLP deployment

QLoRA delivers near-FFT accuracy on commodity GPUs, enabling rapid domain adaptation in resource-constrained clinics. Because the performance gap concentrates in a handful of rare entities, selective fine-tuning or lightweight ensembles could bridge it without retraining the full model. These findings make advanced NLP accessible even to institutions with modest hardware footprints.

### Limitations and future work

Our experiments cover one domain and 2 related languages; behavior in distant language pairs or heterogeneous records remains untested.

Our dataset focuses on multimorbid patients aged over 50 with heart disease or stroke, introducing selection bias and skewed ICD-10 label distributions that limit generalizability.

We also omit domain-adaptive pre-training. Future research should explore the integration of PEFT with medical ontologies or knowledge graphs to improve tail-entity linking.

## Conclusion

This study evaluates PEFT methods as a practical alternative to FFT for multilabel classification of diagnoses in bilingual clinical notes. Working with real-world data written in non-standard Spanish and Catalan, which present unique linguistic challenges not addressed by existing state-of-the-art approaches, the results show that PEFT techniques, particularly QLoRA, can offer competitive performance while substantially reducing computational requirements.

Although FFT still provides the best predictive accuracy, the difference is relatively small when compared with QLoRA. For instance, using BERT trained on a combined Catalan-Spanish corpus, QLoRA achieves a strict Micro-F1 score of 62.2, just 1.27% below the fully fine-tuned model, while requiring 67.5% fewer trainable parameters and reducing memory usage by 33.7%. These savings are especially relevant in clinical settings where access to high-end computing resources may be limited or inconsistent.

Another key finding is the benefit of training on bilingual data. Models trained on the combined corpus consistently generalize better, even when evaluated in a single language. This highlights the value of leveraging multilingual signals in clinical natural language processing, especially in regions with diverse linguistic practices.

Looking ahead, one promising direction is the application of PEFT strategies to large language models (LLMs), which could improve contextual understanding while maintaining manageable resource demands. Combining approaches like QLoRA or LoRA with domain-specific LLMs, along with techniques such as quantization or sparse fine-tuning, may further enhance performance and scalability.

These findings underscore the potential of PEFT methods to enable accurate and efficient clinical NLP systems, particularly in multilingual and resource-constrained healthcare settings.

## Supplementary Material

ooaf120_Supplementary_Data

## Data Availability

Training/validation scripts and 6 best model checkpoints (FFT, LoRA, QLoRA, DoRA, LoHA, and LoKr) are available on DRYAD (DOI: 10.5061/dryad.7m0cfxq8b).^25^
